# Characterization of DNA variants in the human kinome in breast cancer

**DOI:** 10.1038/srep14736

**Published:** 2015-09-30

**Authors:** Divyansh Agarwal, Yuan Qi, Tingting Jiang, Xiuping Liu, Weiwei Shi, Vikram B. Wali, Benjamin Turk, Jeffrey S Ross, W Fraser Symmans, Lajos Pusztai, Christos Hatzis

**Affiliations:** 1 Department of Breast Medical Oncology of Yale University, New Haven, CT, USA; 2 Molecular, Cellular and Developmental Biology of Yale University, New Haven, CT, USA; 3 Department of Quantitative Sciences of the University of Texas M. D. Anderson Cancer Center, Houston, TX, USA; 4 Experimental Therapeutics of the University of Texas M. D. Anderson Cancer Center, Houston, TX, USA; 5 Department of Pharmacology of Yale University, New Haven, CT, USA; 6 Department of Pathology and Laboratory Medicine, Albany Medical College, Albany, NY, USA; 7 Foundation Medicine, Cambridge, MA, USA; 8 Pathology of the University of Texas M. D. Anderson Cancer Center, Houston, TX, USA

## Abstract

Kinases play a key role in cancer biology, and serve as potential clinically useful targets for designing cancer therapies. We examined nucleic acid variations in the human kinome and several known cancer-related genes in breast cancer. DNA was extracted from fine needle biopsies of 73 primary breast cancers and 19 metastatic lesions. Targeted sequencing of 518 kinases and 68 additional cancer related genes was performed using the SOLiD sequencing platform. We detected 1561 unique, non-synonymous variants in kinase genes in the 92 cases, and 74 unique variants in 43 kinases that were predicted to have major functional impact on the protein. Three kinase groups—CMGC, STE and TKL—showed greater mutational load in metastatic compared to primary cancer samples, however, after correction for multiple testing the difference was significant only for the TKL group (*P* = 0.04). We also observed that a higher proportion of histologic grade 1 and 2 cases had high functional impact variants in the SCYL2 gene compared with grade 3 cases. Our findings indicate that individual breast cancers harbor a substantial number of potentially functionally important nucleotide variations in kinase genes, most of which are present in unique combinations and include both somatic and germline functional variants.

The kinome refers to a collection of 518 kinases encoded in the human genome that correspond to approximately 1.7% of the human protein-coding genes^1^. Kinases are important mediators in cell signaling and play key roles in diverse biological processes such as growth, differentiation, metabolism and apoptosis in response to external and internal stimuli. Abnormal activation of kinases can contribute to cancer development and several kinases including B-RAF, MEK, BTK, KIT, ALK, BCR-ABL, EGFR, ERBB2 and VEGFR are clinically useful targets for cancer therapies^2^. Many other investigational drugs are under development, which target various kinases.

Numerous direct and indirect functional interactions exist between kinases and inhibition of a particular kinase may be rendered therapeutically ineffective by compensatory activation of other kinases^3^,^4^,^5^. For instance, in triple negative breast cancer (TNBC), EGFR was suggested as a potential therapeutic target due to its overexpression in these cancers and promising anticancer activity of EGFR inhibitors some cell lines, but clinical trials with EGFR inhibitors failed to show clinically meaningful therapeutic effect, probably due to the existence of alternate compensatory signaling pathways^6^,^7^. Re-routing of signal transduction through alternative pathways in response to MEK inhibitors has also been documented^8^.

Several studies described a variable pattern of kinome gene expression in breast cancer and suggested that kinase expression patterns have prognostic value and could also be used to guide new therapeutic strategies^9^,^10^,^11^. Studies using gene-expression profiling and next-generation sequencing also suggest that breast cancer cell lines express around 375 kinases and there are many potentially functionally important sequence variants present in every cell line^10^,^11^.

The goal of this study was to assess the type and prevalence of potentially functional nucleotide variants (NVs) in the kinome of human breast cancers. We examined if the variants with potential functional impact are uniformly distributed across the kinome or cluster in certain kinase families, and assessed the correlation between variants and different breast cancer subtypes. We did not exclude known, germ line, single nucleotide polymorphisms (SNP) from our analysis because we hypothesized that functional interactions may exist between germ line and somatic events in the kinome. The current study examines the kinase mutation landscape of breast cancer at substantially greater sequencing depth than previous studies, in particular the The Cancer Genome Atlas (TCGA) effort^12^.

## Materials and Methods

### Patients and Samples

Cancer biopsies from 73 patients with primary stage I-III breast cancer who subsequently received neoadjuvant chemotherapy (paclitaxel, 5-fluorouracil, doxorubicin, and cyclophosphamide) and 19 patients with metastatic disease were included in this study. Biopsies were obtained prospectively for biomarker and genomic analysis of cancer in a study approved by the Institutional Review Board at the MD Anderson Cancer Center and all patients signed informed consent. All methods and experiments involving human subjects and tissues were performed in accordance with the institutional guidelines approved by the Yale University School of Medicine and the MD Anderson Cancer Center.

For patients with stage I-III breast cancer, fine needle aspiration of the primary tumor was performed before neoadjuvant chemotherapy and pathologic response was available for all patients and were classified as either pathologic compete response (pCR) or residual cancer (RD). For patients with metastatic cancer, the most safely accessible lesion was biopsied, all patients had multiple prior lines of chemotherapy for metastatic cancer before the biopsy were obtained. Patient characteristics are presented in Table 1. Two needle passes were collected into 1 ml of RNA-later solution (Ambion, Austin, Texas) and stored at −80 °C. DNA was extracted with the QIA-amp DNA Mini Kit (Qiagen, Valencia, California) from the flow-through of a preceding RNA extraction step performed with the RNeasy kit (Qiagen). All biopsies yielded >1.8 μg genomic DNA with 260/280 OD ratio >1.5.

### Kinome sequencing

For target capture and enrichment we used the Agilent SureSelect Human Kinome XT Kit and Target Enrichment system (Supplementary methods). The enrichment system targets approximately 3.2 Mb of the human genome including all known human kinases and a select group of other cancer-related genes and their associated untranslated regions (Supplementary Table 1) (www.agilent.com/genomics/sureselect). SOLiD paired end sequencing kinome multiplexing libraries were constructed from the equal pooled, individual, barcoded kinome library. Batches of 16 barcoded, individual kinome libraries were pooled in equal molar ratio as one multiplexed library and sequenced in one session.

### Variant Calling and Annotation

**Figure 1:**
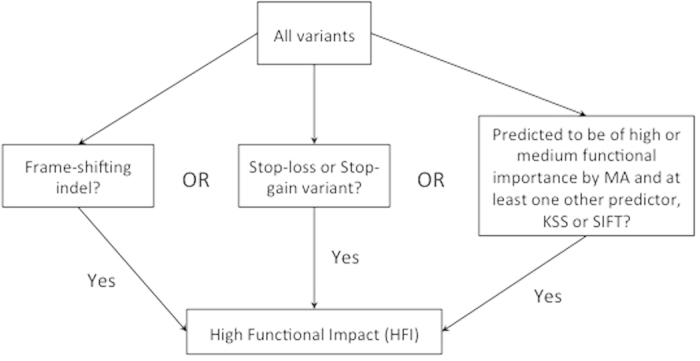
Schematic of the process for determining whether a nucleotide variant is of high functional impact (HFI). An HFI variant (**i**) was predicted to be in the high or medium functional importance category by the Mutation Assessor (MA) and at least one other functional predictor, or (**ii**) it was a stop-gain (nonsense) variant, or (**iii**) a stop-loss variant, or (**iv**) a frame-shifting indel.

All sequenced paired end reads from each barcoded sample were processed by the Applied Biosystems BioScope™ software (version 1.3) using the diBayes and small.indel models to call single nucleotide variants (SNVs) and small insertions or deletions (indels). The reference genome used in this analysis was GRCh37 (hg19). We used ANNOVAR^13^ to annotate the SNVs and indels based on their genomic locations. The functional impact of the variants was determined by using the Mutation Assessor (MA, http://mutationassessor.org/)^14^, a kinase specific functional variant predictor score (KSS) that provides a deleterious versus non deleterious prediction^15^ and by SIFT^16^,^17^. SIFT scores of <0.05 were considered to represent a deleterious variant. A nucleotide variant was called high functional impact (HFI) if (i) it was predicted to be in the high or medium functional importance category by the MA and at least one other predictor, or (ii) it was a stop-gain (nonsense) variant, or (iii) a stop-loss variant, or (iv) a frame-shifting indel (Fig. 1). At the gene level, we called a gene affected, and designated as a variant kinase, if it had at least one nucleotide variant categorized as HFI^15^,^18^. Variants were also annotated by COSMIC and dbSNP/1000 human genome annotation. The number of HFI-variant genes per sample were compared between different breast cancer subtypes and response groups using the Fisher’s exact test. To determine statistical significance, the p-values were adjusted for multiple testing using the Bonferroni correction.

We used the Duplication Gene Database (DGD) to ensure that none of the HFI NVs we found were in co-located or duplicated genes^19^. When calculating the distribution of variant kinases across kinase families in various breast cancer subtypes, gene expression data was used to filter genes that had low expression (i.e. had log 2 normalized values <6.0). The variant kinases were grouped into high expression, and low or absent expression groups to examine if the variant loads differ between the groups. The HFI variant load for each gene was calculated as the number of HFI variants divided by the number of nucleotides in the gene. The 408 expressed kinases were grouped into 10 kinase groups (Supplementary Table 2), and the HFI variant load for each kinase group was calculated as the average variant load of the genes in the group. We also investigated whether certain pairs of somatic mutations and germline variants co-occurred more frequently than other pairs, using the unpaired Wilcoxon rank sum test.

Statistical analysis of the data generated by BioScope was carried out in statistical language R^20^.

### Gene Expression Data

Gene expression data used in this study were generated with Affymetrix HG-U133A gene chips as described before and were previously published (GSE20194)^21^,^22^.

Gene expression array data were normalized using MAS5 with median target array intensity of 600 using R/Bioconductor^23^. Expression values were then log2-transformed. Human protein kinases were identified online (http://kinase.com/human/kinome). We could map 428 of the 529 known kinases to 783 probe sets on the Affymetrix HG-U133A chip using Gene ID annotation (http://www.affymetrix.com/Auth/analysis/downloads/na27/ivt/HG-U133A.na27.annot.csv.zip, version November 30, 2008). When multiple probe sets targeted the same gene, we retained only the probe set with the highest average expression value and greatest variance.

### Validation Cohorts

#### The Cancer Genome Atlas (TCGA) Dataset

We selected all HER2-negative samples from the TCGA dataset that had mutation data available (56 ER-negative and 211 ER-positive). Datasets were downloaded from the TCGA breast carcinoma web site (https://tcga-data.nci.nih.gov/docs/publications/brca_2012/)^12^.

#### Primary-Metastatic Breast Cancer Cohort

We compiled a previously unpublished cohort of 470 breast cancer cases from Foundation Medicine, Inc. This cohort contained 185 metastatic breast cancer samples (39%). The mean age was 54.8 years (standard deviation 12.7) and 49 (10%) of the cancers were *HER2*-amplified. Breast-cancer specific genomic aberrations were profiled in these samples by hybrid capture based targeted sequencing^24^ using the clinically validated Foundation One 315 gene panel (Foundation Medicine, Cambridge, MA). The median coverage for the called aberrations was 546X (5^th^ percentile: 309, 95^th^ percentile: 680).

## Results

### Kinome Sequencing

**Figure 2:**
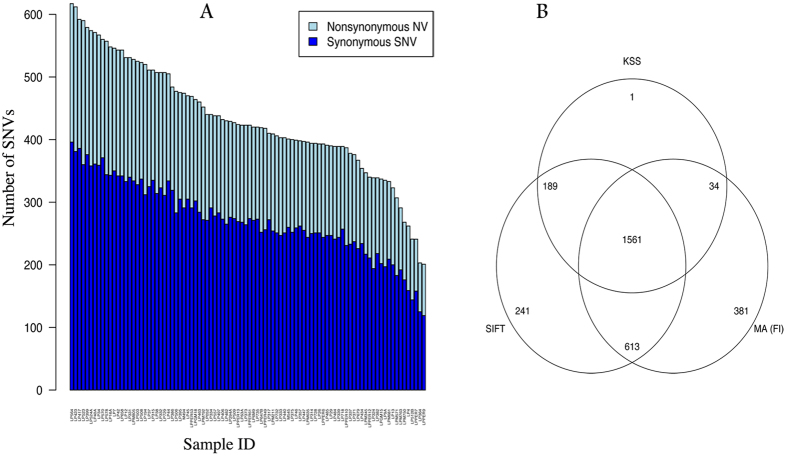
Single nucleotide kinase variants (SNVs) in 92 breast tumors. (**A**) Barplot of number of synonymous, non-synonymous and total SNVs for each of the 92 samples ordered by the total number of SNVs per sample. (**B**) Venn diagram showing the predicted functional impact of 3203 unique non-synonymous SNVs by 3 different functional prediction methods: SIFT scores, Mutation Assessor functional importance (FI) scores, and Kinase Specific functional effect score (KSS). The cutoff used for the three methods to categorize the variants as functionally important were: “high/medium” for Mutation Assessor, <0.05 for SIFT, and “Yes” for KSS deleterious prediction.

Approximately 40 million nucleotide reads were acquired per sample, of which 61% could be mapped to the targeted kinase coding regions, resulting in a mean nucleotide coverage in the targeted regions of 369x. The mean number of nucleotide variants per sample was 1052, ranging from 493 to 1536 in individual cases, 97% of which were already cataloged in dbSNP138 or COSMIC databases. The remaining 3% of the variants were novel. The median number of synonymous variants per sample was 273 (range 119–396) and of non-synonymous variants 153 (range 75–223) (Fig. 2A). The raw data of variants for each patient, limited to only include non-synonymous changes, is provided in Supplementary Table 3.

The three different methods to predict functional importance yielded different numbers of functionally important variants for the same data, but each method predicted that only a small fraction of the detected variants are likely to be functionally important. In all, 3203 unique, non-synonymous variants were detected accross the 92 cases. Of these, 1561 (49%) had a high functional impact score by two or more functional predictors (Fig. 2B). Further filtering by median coverage >20x, we found 295 unique high functional impact (HFI) variants in 148 different genes that were present in at least one of the 92 samples (Supplementary Table 4). One hundred and thirty three of these were known SNPs and 46 were listed in COSMIC. Of the 295 HFI variants, 74 (in 43 kinases) were predicted to have major functional impact on the protein by all 3 functional predictors.

### Technical Reproducibility of Variant Calls

We examined the reproducibility of findings in replicate experiments. Aliquots of the same tumor DNA were sequenced twice in 17 of the samples. The overall concordance rate for all nucleic acid calls was very high ranging from 97 to 99%. However, concordance for variant calls was substantially lower ranging from 75% to 54%. Reproducibility was close to 80% with coverage ≥20x but it did not improve with further increasing coverage above 20x. For all subsequent analysis, therefore we used only variants that had >20 fold coverage and were predicted to be of high functional impact by 2 or more methods (*N* = 295).

We also checked for batch effect using principle component analysis (PCA), and found no evidence for variant detection difference between samples in different sequencing batches. Categorizing the samples based on primary or metastatic tumor origin also yielded similar results, indicating that the number of HFI NVs per gene per sample was not influenced substantially by sequencing batch or sample type (i.e. primary versus metastasis) (Supplementary Figs 1 and 2).

### Distribution of High Functional Impact Variants

**Figure 3:**
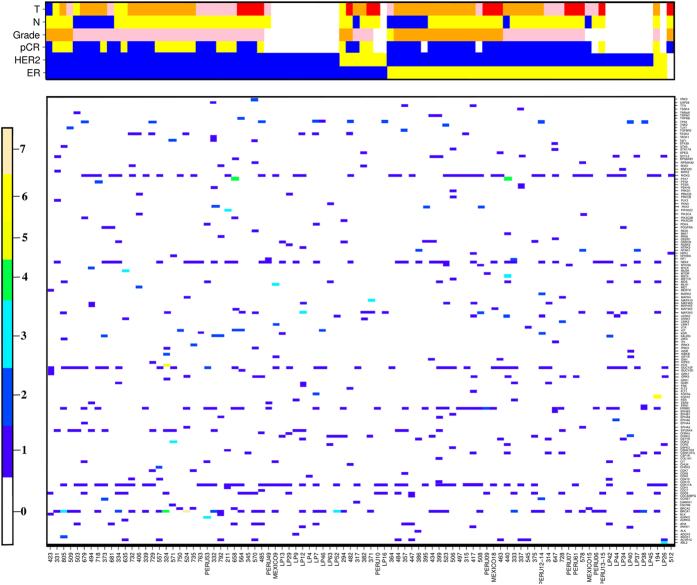
Distribution of high functional-importance (HFI) kinase variants across 92 breast cancers. A total of 295 unique HFI NVs (coverage >20x) in 148 kinase genes are shown at gene level, and the clinical variable status is shown on the top. The samples were ordered hierarchically by ER status, HER2 status, tumor grade, tumor node status (N), tumor size (T-stage), and pathologic complete response (pCR) status (white: missing values for clinical variables, blue: negative; yellow: positive). For Grade and T levels, yellow: 1, orange: 2, pink: 3, red: 4. The legend representing multiple colors, scored from 0–7, in the heatmap correspond to the frequency of HFI variants in a particular gene for a given patient.

The mean number of HFI variants per sample was 24 (range 5 to 40), with each tumor appearing to harbor a unique assortment of variants (Fig. 3). These variants fall into 3 categories: known polymorphisms already reported in dbSNP138; known cancer-associated mutations reported in COSMIC; and low frequency variants with currently unknown role in cancer (Supplementary Table 4). We note that important hotspot mutations in genes like PIK3CA (e.g. E545K and H1047R) and ERBB2 (e.g. L755S) do not get classified as HFI as they were filtered out as low quality calls, and are therefore excluded from the analysis.

The top 10 genes by p-value, with the most frequent HFI variants, are presented in Table 2, and are arranged by ascending p-values. We observed TP53 mutations in 21% the ER negative/HER2 negative cancers and 13% in the ER positive/HER2 negative group. A kinase previously not linked to breast cancer subtypes, TESK2, also showed a trend for more frequently harboring an HFI SNP (rs17853159) in TNBC compared to ER+cancers (16% vs 3%, *P-unadjusted* = 0.06). TESK2 is a dual specificity protein kinase that phosphorylates both serine/threonine and tyrosine residues and has not previously been implicated in cancer but plays a role in spermatogenesis^25^.

We also tested for significant co-occurrence of any pair of known somatic (defined as present in COSMIC) and germ line variants (defined as present in dbSNP) using the Fisher Exact test but observed no significant co-occurrences or mutual exclusions. Classifying the patients according to PAM50, and the variants according to the resulting subtypes, resulted in few mutational load differences at a gene level between the different sub-types, none of which were statistically significant. The small sample size, however, limits the power of these analyses. Comparisons of gene level variant frequencies by response to neoadjuvant therapy and by histologic grade are summarized in Tables 3 and 4, respectively. Although some trends suggesting association with pCR were observed, none reached statistical significance. An HFI SNP in SCYL2 (rs33968174) was seen in 12.5% of grade 1 or 2 cases compared to 0% in grade 3 cases (*P-unadjusted* = 0.04). Of note, SCYL2 is expressed in breast cancer and is involved with clathrin-dependent vesicle trafficking^26^. We compared gene level HFI variant frequencies between TNBC cases who had pCR versus RD but no significant differences in variant distribution was seen.

When we compared HFI variant frequencies across the 10 kinase groups^1^ between metastatic and primary tumors, the CMGC (Cyclin-Dependent and Mitogen-Activated Protein kinases), STE (Ste kinases which then activate the MAPK family) and TKL (Tyrosine-Kinase Like) groups had more functional variants in metastatic cancers compared to primary tumors, however the difference reached statistical significance only for the TKL group (*P* = 0.04). The TK (Tyrosine Kinase) group had a significantly lower mean variant burden in HER2-positive cases compared with triple negative (*P* = 0.01) and ER-positive cancers (*P* = 0.04) (Supplementary Table 5). The kinase group comprising of receptor guanylate cyclases (RGC) was consistently ranked as the group with the highest mean functional variant load but with no significant difference across breast cancer subtypes.

### Mutational Patterns of Kinase Variants in Other Cohorts

We did not find any of the HFI somatic mutations reported in our cohort in the TCGA cancers, likely due to the lower target coverage in TCGA exomes. However, several HFI germline mutations were present at high frequency in the TCGA cohort. A germline mutation in EIF2AK4 (rs35602605) was present in 46% of the triple negative breast cancer (TNBC) cases compared to 27% of the ER-positive/HER2-negative (ER+) cases (*P* = 0.009), confirming the results from our cohort (Table 2). Also a mutation in CDK11A (rs1059831) was present in 96% of the TNBC and 99% of the ER+ in the TCGA cohort. There are not enough metastatic breast cancer cases in the TGCA cohort to evaluate site-specific associations.

**Figure 4:**
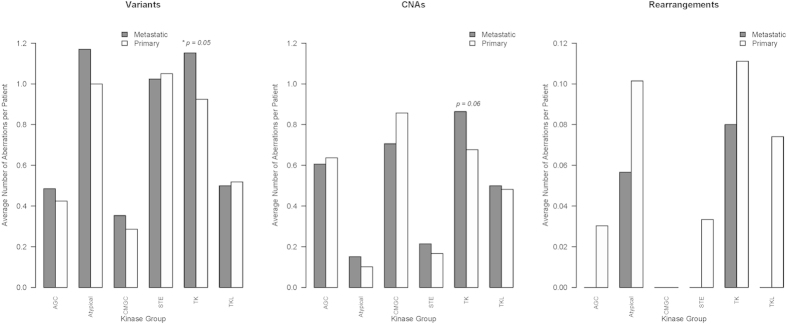
Average number of genomic aberrations per patient from the Foundation Medicine cohort. The first chart shows the results for identified mutations (variants), the second for copy number alterations (CNAs) and the third for genomic rearrangements. Each chart shows the average number of aberrations per patient in patients with metastatic (dark grey) or primary (light grey) tumors occurring in genes within each of the six kinase groups. The p-values are from the comparison of the means in the metastatic and primary groups using a T-test.

We assessed the overall mutational load and potential specificity of kinase mutations in the Foundation Medicine cohort that included mutational profiles from 185 metastatic breast cancers. Among the 49 kinases present in the Foundation One gene panel, genes in the Atypical kinase group (AKT1, AKT2, AKT3, PDK1) had the highest mutation frequently in both primary (88%) and metastatic cancers (96%), followed by genes in the STE kinase group (MAP2K1, MAP2K2, MAP2K4, MAP3K1, PAK3) which were mutated in 83% of the primary and 81% of the metastatic cancers. Genes in the CMCG group (CDK4, CDK6, CDK8, GSK3B) had the most frequent copy number alterations in 86% of the primary and 65% of the metastatic cancers. However, neither difference was statistically significant. Interestingly, both primary and metastatic cancers had at least 1 mutation per patient on average in genes in the Atypical, STE and TK kinase groups (Fig. 4), suggesting that many of these kinases may be important cancer driver genes. Furthermore, about 70–80% of the primary and metastatic tumors appear to have a copy number aberration in genes in CMGC and TK kinase groups (Fig. 4). When comparing the relative mutational load in primary vs metastatic cancers, kinases in the TK group had a borderline significantly greater mutational load in metastatic cancers (*P* = 0.05) and a higher CNA load compared to primary tumors (Fig. 4). Overall these findings are significant and may warrant further investigation.

## Discussion

Understanding the cancer kinome is of interest due to the important regulatory role of these molecules and their potential therapeutic role. The purpose of this analysis was to assess the functional variant landscape of the kinome in breast cancer. We hoped to identify novel high frequency recurrent variants that might represent potential new drug targets. This targeted sequencing study had higher coverage than previous reports including the TCGA; however, we could not identify any novel, high frequency variants. One important observation that we made is that the largest constituent of the high functional impact variants is germline polymorphisms already included in the dbSNP138 and 1000 Human Genomes databases.

In addition to the already known TP53 and PI3K mutations, we detected frequent variants in BRCA1 (20%) and observed several predicted high impact SNVs in many MAPK family enzymes. Predicted HFI SNVs were not distributed evenly across disease subsets; SNVs in ULK4, BMP2K, PALB2, ALPK3 were more frequent in triple negative cancers (TNBC) whereas SNVs in EPHA2 was more common in ER positive cancers. For ER negative patients, we also observed variants in several genes that were not reported in TCGA, including TESK2, EIF2AK4 and CSNK1A1L^12^. An important limitation of our findings is the lack of matching germline DNA for these cases, therefore we cannot reliably distinguish somatic variants from germline events. However, our assumption is that both types of variants maybe functionally relevant.

Another novel implication of our study is the association between variants in SCYL2 with histologic grade, though the association remains weak and statistically insignificant after correction for multiple testing. We observed that a higher proportion of grade 1/2 cases had a functional variant in SCYL2 compared with grade 3 cases. SCYL2 regulates clathrin-dependent vesicle trafficking and contributes to cell migration and invasion^27^. In our study, germline SCYL2 variants are in excess of the numbers predicted based on allele frequency in the 1000 Genomes Project. The minor allele frequency of rs33968174 was 7.6% in our patient cohort, twice that of the 1000 Genomes estimate. When the mutations were categorized into kinase groups, the TKL group had significantly greater mutational load in metastatic cases compared to primary samples, even after the Bonferroni correction *(P* = 0.04). This finding suggests a role for mutations in TKL kinases in cancer metastasis.

Our findings suggest a paucity of shared, high frequency “driver mutations” in kinase genes in breast cancers. Each cancer appears to harbor an individual combination of genomic events that together may constitute the tumor “driver”. Many of the predicted HFI variants are known germ line polymorphisms, which raise the possibility that these may interact with, and could even determine what somatic mutations are functionally important for a given cancer. Depending on the germ line context, different somatic events may constitute cancer driver events in different individuals.

Examining the presumed somatic (in COSMIC) and germline variants (in dbSNP) in individual patients suggests the possibility of functional interactions between the germline and somatic mutations. For example, patient M9 (HER2 negative, ER positive) had a missense germline variant, rs45445894, in the Insulin-like growth factor 1 receptor (IGF1R) and a somatic mutation (c.T1542G; p.C514W) in PI3K catalytic subunit type 2 alpha (PIK3C2A). RNAi-based silencing of PIK3C2A in a large set cancer cell lines showed a critical role for this gene in maintaining the neoplastic phenotype^28^. IGF1R signals through activation of PI3K which also functions as a signal transduction hub for numerous other receptor tyrosine kinases and ultimately regulate several different cellular functions including differentiation, malignant transformation, cell survival and regulating cell–cell adhesion^29^,^30^,^31^. In the patient M9, this critical regulatory pathway is affected at two different levels. Another case, patient LP37 (HER2 negative, ER positive) harbored the missense germline variant rs34584424 in cyclin-dependent kinase 7 (CDK7), which has been shown to alter the active site residues of CDK7 and decrease its binding to kinase inhibitors^32^. CDK7 is a component of both the CDK-activating kinase and the basal transcription factor TFIIH, and can phosphorylate other cyclin-associated kinases CDK1, CDK2, CDK4 and CDK6^33^,^34^. The transcription factor TFIIH is involved in transcription initiation and DNA repair that is necessary for cell cycle progression and is inhibited by p53. Case LP37 also harbored a somatic mutation (c.S199C) in PDK1 which regulates the expression of cyclin D1 and p27 and therefore regulate cell cycle progression (Kip1)^35^. Alterations in both genes might imply a cumulative effect on dysregulation of cell proliferation.

While the somatic-germline hypothesis might be a possibility, that kinases that need not be mutated may be activated by overexpression, and might also be able to explain the paucity of kinase variants. Our results are largely observational and do not point to new recurrent HFI variants; however, overall, our study provides supportive evidence for potentially unique combinations of kinase and other gene variants in breast cancer. The variants of unknown significance identified here would need to be validated externally and by functional assays to strengthen the claim for their functional significance.

In conclusion, we found that individual breast cancers harbor a large number of potentially functionally important nucleotide variations in kinase genes (the mean number of kinase variants was 24 per case). Most of the HFI SNVs include previously described germline variants with experimentally validated or suspected impact on protein function. Individual variants are rare and are present in unique combinations in each case. We hypothesize that any particular somatic mutation may cause different biological consequences depending on the constellation of functionally active germline SNPs that are present in the same cell. An important future challenge is to experimentally assess the functional interaction between germline and somatic variants and determine how, if at all, they contribute to the biological behavior of cancer.

## Additional Information

**How to cite this article**: Agarwal, D. *et al.* Characterization of DNA variants in the human kinome in breast cancer. *Sci. Rep.* **5**, 14736; doi: 10.1038/srep14736 (2015).

## Supplementary Material

Supplementary Material

Supplementary Table 3

Supplementary Table 4

## Figures and Tables

**Table 1:** Summary of Clinical Characteristics of the 92 Samples Used in the Study.

Characteristics	# Patients	%
Age at diagnosis		
Median	50.5	
Range	28–86	
T-stage at diagnosis		
T0	2	2
T1	9	10
T2	42	45
T3	17	18
T4	20	22
Unknown	2	2
N-grade at diagnosis		
N0	22	24
N1 to N3	69	74
Unknown	1	1
AJCC stage at diagnosis		
I	4	4
II	37	40
III	44	47
IV	6	6
Unknown	1	1
ER status		
Positive	44	47
Negative	47	51
Uncertain	1	1
PR status		
Positive	33	35
Negative	58	62
Unknown	1	1
HER2 status		
Positive	2	2
Negative	54	58
Unknown	36	39
Nuclear grade		
1	1	1
2	27	29
3	56	60
Unknown	8	9

**Table 2:** Number and percentage of gene-level high functional impact variants by Estrogen Receptor (ER) Status.

Gene	ER-negative/HER2-negative (N = 43) (%)	ER-positive/HER2-negative (N = 39) (%)	Fisher’s Exact Test P-value
TESK2	7 (16.3%)	1 (2.6%)	0.06
EIF2AK4	11 (25.6%)	4 (10.3%)	0.09
NTRK1	0 (0%)	3 (7.7%)	0.10
RIOK2	15 (34.9%)	21 (53.8%)	0.12
PTEN	0 (0%)	1 (2.5%)	0.18
DSTYK	1 (2.3%)	4 (10.3%)	0.19
SCYL2	1 (2.3%)	4 (10.3%)	0.19
CDK11A	24 (55.8%)	16 (41.0%)	0.19
NPR1	0 (0%)	2 (5.1%)	0.22
CSNK1A1L	2 (4.7%)	5 (12.8%)	0.25

The P-values reported are unadjusted for multiple testing. If there was at least one HFI NV in a gene in a sample, that gene was considered as HFI variant. Only the 10 most frequently observed variant genes are shown, ordered by p-values.

**Table 3:** High functional impact gene variant frequencies between response groups of triple negative breast cancer (pCR = pathologic complete response, RD = residual disease after preoperative chemotherapy).

Gene	pCR (N = 18) (%	RD (N = 19) (%)	Fisher’s Exact Test P-value
CDK11A	6 (33.3%)	12 (63.2%)	0.10
BRCA2	3 (16.7%)	0 (0%)	0.11
KALRN	4 (22.2%)	1 (5.3%)	0.18
BRCA1	13 (72.2%)	9 (47.4%)	0.18
IDH1	2 (11.1%)	0 (0%)	0.23
CDC6	2 (11.1%)	6 (31.6%)	0.23
NEK4	8 (44.4%)	5 (26.3%)	0.31
CIT	0 (0%)	2 (10.5%)	0.48
GUCY2D	0 (0%)	2 (10.5%)	0.48
PTK2	0 (0%)	2 (10.5%)	0.48

The P-values reported are unadjusted for multiple testing. If there was at least one HFI NV in a gene in a sample, that gene was considered high functional impact variant. Only the 10 most frequently observed variant genes are shown, ordered by p-values.

**Table 4:** High functional impact gene variant frequencies by histologic grade.

Gene	Grade 1/2 (N = 24) (%)	Grade 3 (N = 44) (%)	Fisher’s Exact Test P-value
SCYL2	3 (12.5%)	0 (0%)	0.04
TRPM7	2 (8.3%)	0 (0%)	0.12
PKN1	4 (16.7%)	2 (4.5%)	0.17
DSTYK	2 (8.3%)	1 (2.3%)	0.28
MYO3A	2 (8.3%)	1 (2.3%)	0.28
GAB1	0 (0%)	4 (9.1%)	0.29
RIOK2	13 (54.2%)	18 (40.1%)	0.32
MTOR	1 (4.2%)	0 (0%)	0.35
PRKD1	1 (4.2%)	0 (0%)	0.35
ADCK1	1 (4.2%)	0 (0%)	0.35

The P-values reported are unadjusted for multiple testing. If there was at least one HFI NV in a gene in a sample, that gene was considered high functional impact variant. Only the 10 most frequently observed variant genes are shown, ordered by p-values.
