# Supplementary material for: Characterization of DNA variants in the human kinome in breast cancer
Source: Sci Rep. 2015 Sep 30;5:14736. doi: 10.1038/srep14736 (PMC4588561; doi:10.1038/srep14736)
Supplement: Supplementary Material [file srep14736-s1.doc]

**Characterization of DNA variants in the human kinome in breast cancer**

Divyansh Agarwal^a,b^, Yuan Qi^c^, Tingting Jiang^a^, Xiuping Liu^d^, Weiwei Shi^a^, Vikram B. Wali^a^, Benjamin Turk^e^, Jeffrey S Ross^f, g^, W Fraser Symmans^h^, Lajos Pusztai^a^, Christos Hatzis^a^*

^a^Department of Breast Medical Oncology, ^b^Molecular, Cellular and Developmental Biology, and ^e^Department of Pharmacology of Yale University, New Haven, CT, U.S.A.

^c^Department of Quantitative Sciences, ^d^Experimental Therapeutics, and ^h^Pathology of the University of Texas M. D. Anderson Cancer Center, Houston, TX, U.S.A.

^f^Department of Pathology and Laboratory Medicine, Albany Medical College, Albany, NY, U.S.A

^g^Foundation Medicine, Cambridge, MA, U.S.A.

*** Address correspondence to:**

Christos Hatzis, Ph.D., Yale Comprehensive Cancer Center, Yale School of Medicine, New Haven CT 06520, U.S.A.

E-mail: [christos.hatzis@yale.edu](mailto:christos.hatzis@yale.edu)

Table of Contents (**Supplementary Data**)

Supplementary Methods 4

Details about patients and target enrichment for kinome sequencing.

Kinome Sequencing Methods 4

**Target Enrichment for Kinome Sequencing 4**

Supplementary Figure 1 6

Principle component analysis (PCA) plot of the number of HFI NVs detected simultaneously in a gene in a sample by neoadjuvant (red) or metastatic sample (black).

Supplementary Figure 2 7

Principle component analysis (PCA) plot of the number of HFI NVs detected simultaneously in a gene in a sample by sequencing batch. The samples were colored by sequencing batch (slides).

Supplementary Table 1 8

List of the genes captured in the kinome sequencing of 92 breast cancer patients using the SOLiD platform. Key: Red -Kinase Genes; Blue - PIK3 Domain Proteins; Green - Diglyceride Kinases; Pink - Cancer Genes; Orange - PIK3 Regulatory Components; Black - Additional Breast Cancer Genes

Supplementary Table 2 27

List of the 408 expressed kinase genes and their assignment into 10 kinase families.

Supplementary Table 3 (Supplemental Table 3 Raw Data 12-3-14) SEE ATTACHED

Raw data of the list of variants for each patient, annotated with the functional score and median coverage.

Supplementary Table 4 (Supplemental Table 4 Variants 12-3-14) ATTACHED

Unique HFI variants (n=307) with a median coverage > 20x, present in 142 different genes in at least one of the 92 samples. ). The variants are annotated to indicate whether the polymorphisms are already reported in dbSNP138, or COSMIC. Those lacking annotation in either field are the low frequency variants in genes with currently unknown role in cancer.

Supplementary Table 5 41

Results for comparison of the mean mutational load per patient per gene between 10 kinase groups. All p-values were computed using an unpaired Wilcoxon test. Patients were categorized into different subtypes, and the mutation data was filtered for expressed kinases and predicted HFI.

# Supplementary Methods

***Kinome Sequencing Methods***

DNA aberrations in the whole kinome were determined using the SureSelect XT Target Enrichment Kits with AB SOLiD Multiplexed Sequencing (Version 1.0, Nov.2010). In brief, 1.8-3.0 µg of genomic DNA from each sample was fragmented into peak fragment size of 150-180 base pairs (Covaris S2 instrument, Covaris Inc. Woburn, Massachusetts). Fragment purification was performed with Agenecourt AMPure plus beads (Beckman, P/N A63881) followed by end repair using T4 DNA polymerase and Klenow DNA polymerase at room temperature for 30 minutes. The purified, end-repaired fragments were ligated with P1 and 1A adaptors on both ends at room temperature for 15 minutes. Subsequently, 200bp DNA fragments with ligated adaptors were isolated by electrophoresis using E-gel SizeSelect 2% gel (Invitrogen, P/N G661002) and were amplified by nick translation performed on PCR 9700 thermocycler using SureSelect pre-capture primers x 12 cycles. The PCR products were purified and quantified by Agilent bioanalyzer 2100 DNA 1000 assay. The expected size distribution of this amplified genomic DNA library with P1 and truncated multiplex P2 adaptors is 250-275bp.

***Target enrichment for kinome sequencing***

Five hundred ng of DNA from individual genomic libraries was hybridized with SureSelect kinome capture library, which is a mixture of 120 nucleotide-long biotinylated RNA baits used as probes for 612 target genes designed from 10,282 exons, following the manufacturer's instructions. After 24 hours of hybridization at 65˚C, the target regions were isolated by pulling down the biotinylated probe/target hybrids with streptavidin-coated magnetic beads (Dynal MyOne Streptavidin T1, Invitrogen). The captured target regions were purified using Agenecourt AMPure beads. The target DNA libraries from each replicate were enriched and multiplexing barcodes were added through a 9-cycle PCR amplification step using the SureSelect SOLiD barcode multiplexing PCR primer set. The PCR products representing the final individual, enriched, kinome sequencing library were purified by Agenecourt AMPure Plus beads and quantitated with Agilent Bioanalyzer 2100; the expected size distribution of the final target library is 270 - 350 base pairs.

# Supplementary Figure 1: Principle component analysis (PCA) plot of the number of HFI NVs detected simultaneously in a gene in a sample by neoadjuvant (red) or metastatic sample (black).


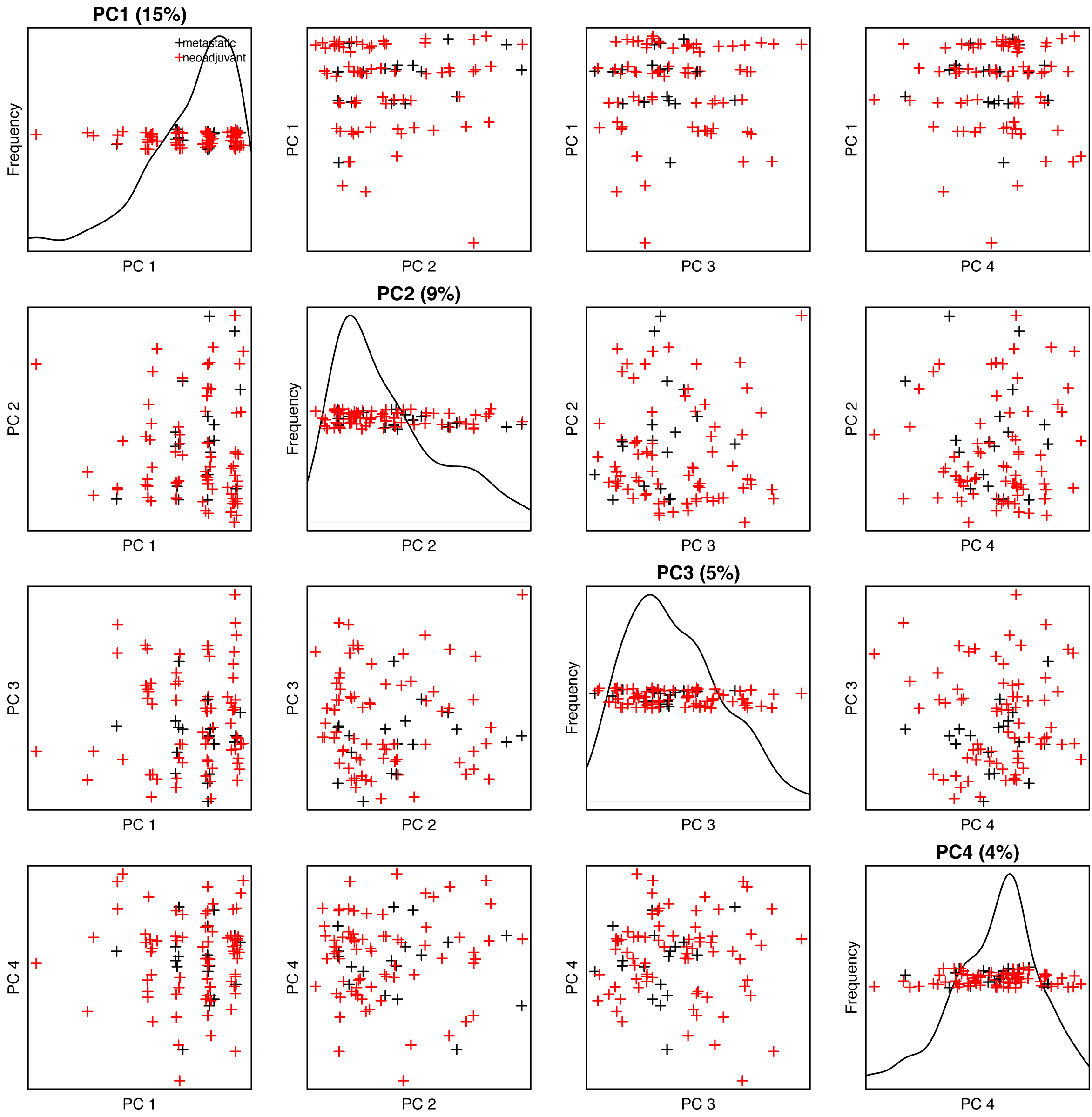


# Supplementary Figure 2: Principle component analysis (PCA) plot of the number of HFI NVs detected simultaneously in a gene in a sample, by sequencing batch. The samples were colored by sequencing batch (slides).

#
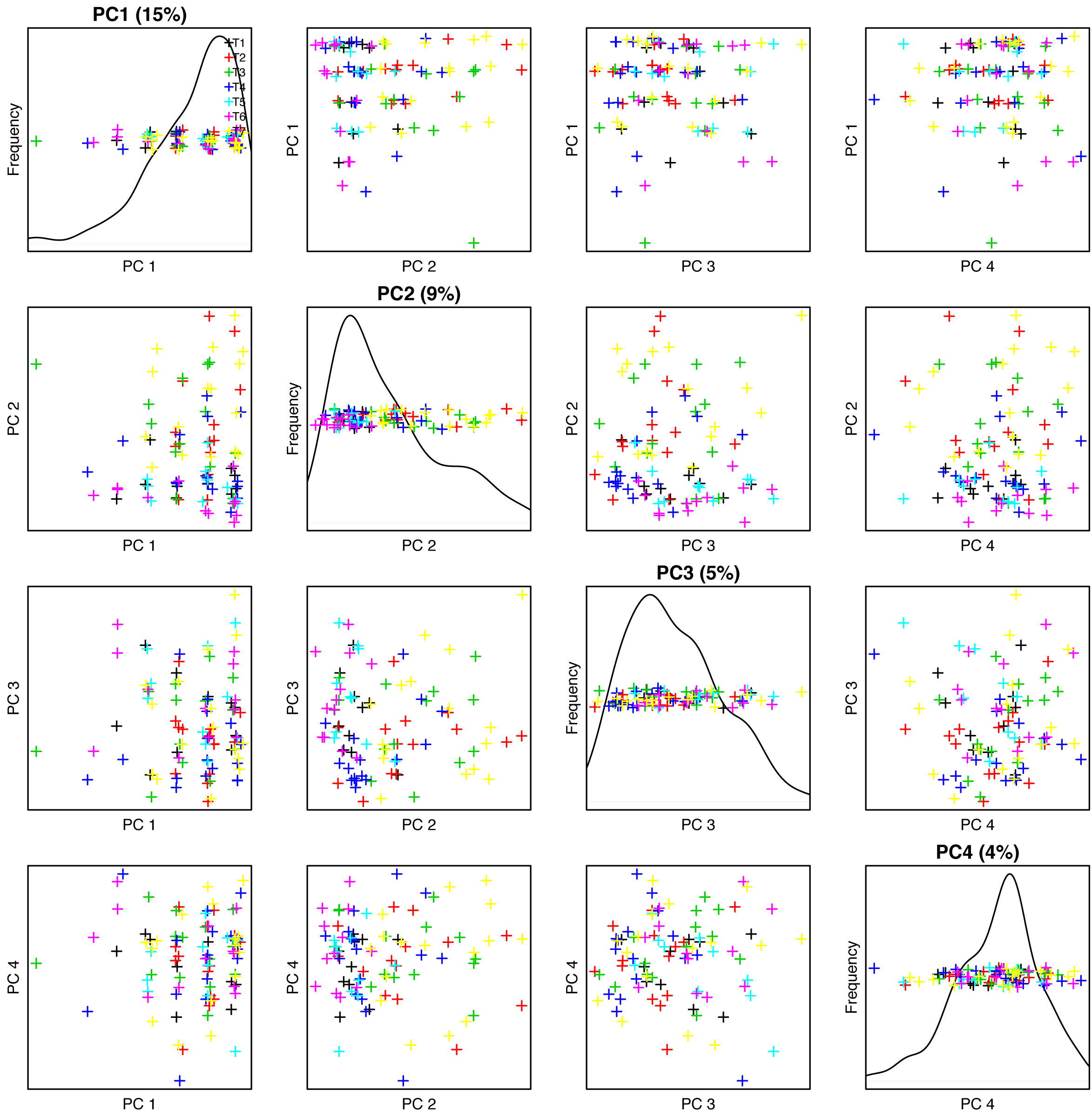


# Supplementary Table 1: List of the genes captured in the kinome sequencing of 92 breast cancer patients using the SOLiD platform.

| **Gene_Symbol** | **UniGene_ID** |
| --- | --- |
| AAK1 | Hs.468878 |
| AATK | Hs.514575 |
| ABL1 | Hs.431048 |
| ABL2 | Hs.159472 |
| ACTR2 | Hs.719274 |
| ACVR1 | Hs.470316 |
| ACVR1B | Hs.438918 |
| ACVR1C | Hs.562901 |
| ACVR2A | Hs.470174 |
| ACVR2B | Hs.174273 |
| ACVRL1 | Hs.591026 |
| ADCK1 | Hs.413208 |
| ADCK4 | Hs.130712 |
| ADCK5 | Hs.283374 |
| ADRBK1 | Hs.83636 |
| ADRBK2 | Hs.657494 |
| AKT1 | Hs.525622 |
| AKT2 | Hs.631535 |
| AKT3 | Hs.498292 |
| ALK | Hs.654469 |
| ALPK1 | Hs.652825 |
| ALPK2 | Hs.628152 |
| ALPK3 | Hs.459183 |
| AMHR2 | Hs.659889 |
| ANKK1 | Hs.448473 |
| ARAF | Hs.446641 |
| ATM | Hs.367437 |
| ATR | Hs.271791 |
| AURKA | Hs.250822 |
| AURKB | Hs.442658 |
| AURKC | Hs.98338 |
| AXL | Hs.590970 |
| BCKDK | Hs.513520 |
| BLK | Hs.146591 |
| BMP2K | Hs.146551 |
| BMPR1A | Hs.524477 |
| BMPR1B | Hs.598475 |
| BMPR2 | Hs.471119 |
| BMX | Hs.495731 |
| BRAF | Hs.550061 |
| BRD2 | Hs.75243 |
| BRD3 | Hs.522472 |
| BRD4 | Hs.187763 |
| BRDT | Hs.482520 |
| BRSK1 | Hs.182081 |
| BRSK2 | Hs.170819 |
| BTK | Hs.159494 |
| BUB1 | Hs.469649 |
| BUB1B | Hs.631699 |
| C9orf96 | Hs.159448 |
| CABC1 | Hs.118241 |
| CAMK1 | Hs.434875 |
| CAMK1D | Hs.659517 |
| CAMK1G | Hs.199068 |
| CAMK2A | Hs.716391 |
| CAMK2B | Hs.351887 |
| CAMK2D | Hs.144114 |
| CAMK2G | Hs.523045 |
| CAMK4 | Hs.591269 |
| CAMKK1 | Hs.8417 |
| CAMKK2 | Hs.297343 |
| CAMKV | Hs.145156 |
| CASK | Hs.495984 |
| CCRK | Hs.522274 |
| CDC2 | Hs.334562 |
| CDC2L2 | Hs.709182 |
| CDC2L5 | Hs.233552 |
| CDC2L6 | Hs.719138 |
| CDC42BPA | Hs.35433 |
| CDC42BPB | Hs.654634 |
| CDC42BPG | Hs.293590 |
| CDC7 | Hs.533573 |
| CDK10 | Hs.699177 |
| CDK2 | Hs.19192 |
| CDK3 | Hs.706766 |
| CDK4 | Hs.95577 |
| CDK5 | Hs.647078 |
| CDK6 | Hs.119882 |
| CDK7 | Hs.184298 |
| CDK8 | Hs.382306 |
| CDK9 | Hs.150423 |
| CDKL1 | Hs.679430 |
| CDKL2 | Hs.591698 |
| CDKL3 | Hs.105818 |
| CDKL4 | Hs.403201 |
| CDKL5 | Hs.659851 |
| CHEK1 | Hs.24529 |
| CHEK2 | Hs.291363 |
| CHUK | Hs.198998 |
| CIT | Hs.119594 |
| CLK1 | Hs.433732 |
| CLK2 | Hs.73986 |
| CLK3 | Hs.584748 |
| CLK4 | Hs.406557 |
| CNKSR2 | Hs.555917 |
| CRKRS | Hs.416108 |
| CSF1R | Hs.586219 |
| CSK | Hs.77793 |
| CSNK1A1 | Hs.712555 |
| CSNK1A1L | Hs.512897 |
| CSNK1D | Hs.631725 |
| CSNK1E | Hs.474833 |
| CSNK1G1 | Hs.646508 |
| CSNK1G2 | Hs.651905 |
| CSNK1G3 | Hs.129206 |
| CSNK2A1 | Hs.644056 |
| CSNK2A2 | Hs.82201 |
| DAPK1 | Hs.380277 |
| DAPK2 | Hs.237886 |
| DAPK3 | Hs.631844 |
| DCLK1 | Hs.507755 |
| DCLK2 | Hs.591683 |
| DCLK3 | Hs.631907 |
| DDR1 | Hs.631988 |
| DDR2 | Hs.593833 |
| DMPK | Hs.631596 |
| DSTYK | Hs.6874 |
| DYRK1A | Hs.719269 |
| DYRK1B | Hs.130988 |
| DYRK2 | Hs.173135 |
| DYRK3 | Hs.164267 |
| DYRK4 | Hs.439530 |
| EEF2K | Hs.498892 |
| EGFR | Hs.488293 |
| EIF2AK1 | Hs.719136 |
| EIF2AK2 | Hs.131431 |
| EIF2AK3 | Hs.591589 |
| EIF2AK4 | Hs.656673 |
| EPHA1 | Hs.89839 |
| EPHA10 | Hs.129435 |
| EPHA2 | Hs.171596 |
| EPHA3 | Hs.123642 |
| EPHA4 | Hs.371218 |
| EPHA5 | Hs.654492 |
| EPHA6 | Hs.653244 |
| EPHA7 | Hs.73962 |
| EPHA8 | Hs.283613 |
| EPHB1 | Hs.116092 |
| EPHB2 | Hs.523329 |
| EPHB3 | Hs.2913 |
| EPHB4 | Hs.437008 |
| EPHB6 | Hs.380089 |
| ERBB2 | Hs.446352 |
| ERBB3 | Hs.118681 |
| ERBB4 | Hs.390729 |
| ERN1 | Hs.700027 |
| ERN2 | Hs.592041 |
| FASTK | Hs.647094 |
| FER | Hs.221472 |
| FES | Hs.7636 |
| FGFR1 | Hs.264887 |
| FGFR2 | Hs.533683 |
| FGFR3 | Hs.1420 |
| FGFR4 | Hs.165950 |
| FGR | Hs.1422 |
| FLJ25006 | Hs.657973 |
| FLT1 | Hs.654360 |
| FLT3 | Hs.507590 |
| FLT4 | Hs.646917 |
| FRK | Hs.89426 |
| FYN | Hs.390567 |
| GAK | Hs.369607 |
| GCK | Hs.1270 |
| GRK1 | Hs.103501 |
| GRK4 | Hs.32959 |
| GRK5 | Hs.524625 |
| GRK6 | Hs.235116 |
| GRK7 | Hs.680654 |
| GSG2 | Hs.534059 |
| GSK3A | Hs.466828 |
| GSK3B | Hs.445733 |
| GUCY2C | Hs.524278 |
| GUCY2D | Hs.592109 |
| GUCY2F | Hs.123074 |
| HCK | Hs.655210 |
| HIPK1 | Hs.532363 |
| HIPK2 | Hs.397465 |
| HIPK3 | Hs.201918 |
| HIPK4 | Hs.79363 |
| HSPB8 | Hs.400095 |
| HUNK | Hs.109437 |
| ICK | Hs.417022 |
| IGF1R | Hs.643120 |
| IKBKB | Hs.597664 |
| IKBKE | Hs.321045 |
| ILK | Hs.706355 |
| INSR | Hs.465744 |
| INSRR | Hs.248138 |
| IRAK1 | Hs.522819 |
| IRAK2 | Hs.449207 |
| IRAK3 | Hs.369265 |
| IRAK4 | Hs.138499 |
| ITK | Hs.558348 |
| JAK1 | Hs.207538 |
| JAK2 | Hs.656213 |
| JAK3 | Hs.515247 |
| KALRN | Hs.8004 |
| KDR | Hs.479756 |
| KIAA1804 | Hs.547779 |
| KIT | Hs.479754 |
| KSR1 | Hs.133534 |
| KSR2 | Hs.375836 |
| LATS1 | Hs.716697 |
| LATS2 | Hs.78960 |
| LCK | Hs.470627 |
| LIMK1 | Hs.647035 |
| LIMK2 | Hs.474596 |
| LMTK2 | Hs.444179 |
| LMTK3 | Hs.207426 |
| LRRK1 | Hs.407918 |
| LRRK2 | Hs.187636 |
| LTK | Hs.434481 |
| LYN | Hs.699154 |
| MAK | Hs.446125 |
| MAP2K1 | Hs.145442 |
| MAP2K2 | Hs.465627 |
| MAP2K3 | Hs.514012 |
| MAP2K4 | Hs.514681 |
| MAP2K5 | Hs.114198 |
| MAP2K6 | Hs.463978 |
| MAP2K7 | Hs.531754 |
| MAP3K1 | Hs.653654 |
| MAP3K10 | Hs.466743 |
| MAP3K11 | Hs.502872 |
| MAP3K12 | Hs.713539 |
| MAP3K13 | Hs.656069 |
| MAP3K14 | Hs.404183 |
| MAP3K15 | Hs.713701 |
| MAP3K2 | Hs.145605 |
| MAP3K3 | Hs.29282 |
| MAP3K4 | Hs.390428 |
| MAP3K5 | Hs.186486 |
| MAP3K6 | Hs.194694 |
| MAP3K7 | Hs.719192 |
| MAP3K8 | Hs.432453 |
| MAP3K9 | Hs.593542 |
| MAP4K1 | Hs.95424 |
| MAP4K2 | Hs.534341 |
| MAP4K3 | Hs.655750 |
| MAP4K4 | Hs.719073 |
| MAP4K5 | Hs.130491 |
| MAPK1 | Hs.431850 |
| MAPK10 | Hs.125503 |
| MAPK11 | Hs.57732 |
| MAPK12 | Hs.432642 |
| MAPK13 | Hs.178695 |
| MAPK14 | Hs.485233 |
| MAPK15 | Hs.493169 |
| MAPK3 | Hs.861 |
| MAPK4 | Hs.433728 |
| MAPK6 | Hs.411847 |
| MAPK7 | Hs.150136 |
| MAPK8 | Hs.138211 |
| MAPK9 | Hs.484371 |
| MAPKAPK2 | Hs.643566 |
| MAPKAPK3 | Hs.234521 |
| MAPKAPK5 | Hs.413901 |
| MARK1 | Hs.497806 |
| MARK2 | Hs.567261 |
| MARK3 | Hs.35828 |
| MARK4 | Hs.34314 |
| MAST1 | Hs.227489 |
| MAST2 | Hs.319481 |
| MAST3 | Hs.466184 |
| MAST4 | Hs.595458 |
| MASTL | Hs.276905 |
| MATK | Hs.631845 |
| MELK | Hs.184339 |
| MERTK | Hs.306178 |
| MET | Hs.132966 |
| MGC42105 | Hs.25845 |
| MINK1 | Hs.443417 |
| MKNK1 | Hs.371594 |
| MKNK2 | Hs.515032 |
| MLKL | Hs.119878 |
| MOS | Hs.533432 |
| MST1R | Hs.517973 |
| MTOR | Hs.338207 |
| MUSK | Hs.521653 |
| MYLK | Hs.477375 |
| MYLK2 | Hs.86092 |
| MYLK3 | Hs.130465 |
| MYLK4 | Hs.127830 |
| MYO3A | Hs.662630 |
| MYO3B | Hs.671900 |
| NEK1 | Hs.481181 |
| NEK10 | Hs.506115 |
| NEK11 | Hs.657336 |
| NEK2 | Hs.153704 |
| NEK3 | Hs.409989 |
| NEK4 | Hs.631921 |
| NEK5 | Hs.672144 |
| NEK6 | Hs.197071 |
| NEK7 | Hs.24119 |
| NEK8 | Hs.448468 |
| NEK9 | Hs.719118 |
| NLK | Hs.208759 |
| NPR1 | Hs.490330 |
| NPR2 | Hs.78518 |
| NRBP1 | Hs.515876 |
| NRBP2 | Hs.521926 |
| NRK | Hs.209527 |
| NTRK1 | Hs.406293 |
| NTRK2 | Hs.494312 |
| NTRK3 | Hs.410969 |
| NUAK1 | Hs.719171 |
| NUAK2 | Hs.497512 |
| OBSCN | Hs.656999 |
| OXSR1 | Hs.475970 |
| PAK1 | Hs.435714 |
| PAK2 | Hs.518530 |
| PAK3 | Hs.656789 |
| PAK4 | Hs.20447 |
| PAK6 | Hs.513645 |
| PAK7 | Hs.32539 |
| PASK | Hs.397891 |
| PBK | Hs.104741 |
| PCTK1 | Hs.496068 |
| PCTK2 | Hs.506415 |
| PCTK3 | Hs.445402 |
| PDGFRA | Hs.74615 |
| PDGFRB | Hs.509067 |
| PDIK1L | Hs.468801 |
| PDK1 | Hs.470633 |
| PDK2 | Hs.256667 |
| PDK3 | Hs.658190 |
| PDK4 | Hs.8364 |
| PDPK1 | Hs.459691 |
| PFTK1 | Hs.430742 |
| PFTK2 | Hs.348711 |
| PHKG1 | Hs.715728 |
| PHKG2 | Hs.196177 |
| PIK3R4 | Hs.149032 |
| PIM1 | Hs.81170 |
| PIM2 | Hs.719294 |
| PIM3 | Hs.530381 |
| PINK1 | Hs.389171 |
| PKLR | Hs.95990 |
| PKMYT1 | Hs.77783 |
| PKN1 | Hs.466044 |
| PKN2 | Hs.440833 |
| PKN3 | Hs.300485 |
| PLK1 | Hs.592049 |
| PLK2 | Hs.398157 |
| PLK3 | Hs.632415 |
| PLK4 | Hs.172052 |
| PNCK | Hs.436667 |
| PRAGMIN | Hs.657673 |
| PRKAA1 | Hs.43322 |
| PRKAA2 | Hs.437039 |
| PRKACA | Hs.631630 |
| PRKACB | Hs.487325 |
| PRKACG | Hs.158029 |
| PRKCA | Hs.531704 |
| PRKCB | Hs.460355 |
| PRKCD | Hs.155342 |
| PRKCE | Hs.580351 |
| PRKCG | Hs.631564 |
| PRKCH | Hs.333907 |
| PRKCI | Hs.478199 |
| PRKCQ | Hs.498570 |
| PRKCZ | Hs.496255 |
| PRKD1 | Hs.508999 |
| PRKD2 | Hs.466987 |
| PRKD3 | Hs.660757 |
| PRKDC | Hs.491682 |
| PRKG1 | Hs.654556 |
| PRKG2 | Hs.570833 |
| PRKX | Hs.390788 |
| PRKY | Hs.632287 |
| PRPF4B | Hs.159014 |
| PSKH1 | Hs.513683 |
| PSKH2 | Hs.680136 |
| PTK2 | Hs.395482 |
| PTK2B | Hs.491322 |
| PTK6 | Hs.51133 |
| PTK7 | Hs.90572 |
| PXK | Hs.190544 |
| RAC1 | Hs.413812 |
| RAF1 | Hs.159130 |
| RAGE | Hs.104119 |
| RET | Hs.350321 |
| RIOK1 | Hs.437474 |
| RIOK2 | Hs.27021 |
| RIOK3 | Hs.719109 |
| RIPK1 | Hs.519842 |
| RIPK2 | Hs.103755 |
| RIPK3 | Hs.268551 |
| RIPK4 | Hs.517310 |
| RNASEL | Hs.518545 |
| ROCK1 | Hs.306307 |
| ROCK2 | Hs.591600 |
| ROR1 | Hs.654491 |
| ROR2 | Hs.98255 |
| ROS1 | Hs.1041 |
| RP6-213H19.1 | Hs.444247 |
| RPS6KA1 | Hs.149957 |
| RPS6KA2 | Hs.719131 |
| RPS6KA3 | Hs.445387 |
| RPS6KA4 | Hs.105584 |
| RPS6KA5 | Hs.510225 |
| RPS6KA6 | Hs.368153 |
| RPS6KB1 | Hs.463642 |
| RPS6KB2 | Hs.534345 |
| RPS6KC1 | Hs.591416 |
| RPS6KL1 | Hs.414481 |
| RYK | Hs.654562 |
| SBK1 | Hs.97837 |
| SBK2 | Hs.532676 |
| SCYL1 | Hs.238839 |
| SCYL2 | Hs.506481 |
| SCYL3 | Hs.435560 |
| SGK1 | Hs.510078 |
| SGK196 | Hs.491646 |
| SGK2 | Hs.300863 |
| SGK269 | Hs.9587 |
| SGK3 | Hs.613417 |
| SGK493 | Hs.408542 |
| SIK1 | Hs.282113 |
| SIK2 | Hs.269128 |
| SIK3 | Hs.167451 |
| SLK | Hs.591922 |
| SMG1 | Hs.460179 |
| SNRK | Hs.476052 |
| SPEG | Hs.21639 |
| SRC | Hs.195659 |
| SRM | Hs.76244 |
| SRMS | Hs.411061 |
| SRPK1 | Hs.443861 |
| SRPK2 | Hs.285197 |
| SRPK3 | Hs.104865 |
| STK10 | Hs.719134 |
| STK11 | Hs.515005 |
| STK16 | Hs.153003 |
| STK17A | Hs.709489 |
| STK17B | Hs.88297 |
| STK19 | Hs.654371 |
| STK24 | Hs.508514 |
| STK25 | Hs.516807 |
| STK3 | Hs.492333 |
| STK31 | Hs.309767 |
| STK32A | Hs.585069 |
| STK32B | Hs.133062 |
| STK32C | Hs.469002 |
| STK33 | Hs.501833 |
| STK35 | Hs.100057 |
| STK36 | Hs.471404 |
| STK38 | Hs.409578 |
| STK38L | Hs.184523 |
| STK39 | Hs.276271 |
| STK4 | Hs.472838 |
| STK40 | Hs.471768 |
| STRADA | Hs.514402 |
| STRADB | Hs.652338 |
| STYK1 | Hs.24979 |
| SYK | Hs.371720 |
| TAF1 | Hs.158560 |
| TAF1L | Hs.591086 |
| TAOK1 | Hs.631758 |
| TAOK2 | Hs.291623 |
| TAOK3 | Hs.644420 |
| TBCK | Hs.292986 |
| TBK1 | Hs.505874 |
| TEC | Hs.479670 |
| TEK | Hs.89640 |
| TESK1 | Hs.708096 |
| TESK2 | Hs.591499 |
| TEX14 | Hs.390221 |
| TGFBR1 | Hs.494622 |
| TGFBR2 | Hs.82028 |
| TIE1 | Hs.78824 |
| TLK1 | Hs.719163 |
| TLK2 | Hs.445078 |
| TNIK | Hs.34024 |
| TNK1 | Hs.203420 |
| TNK2 | Hs.518513 |
| TNNI3K | Hs.480085 |
| TP53RK | Hs.440263 |
| TRIB1 | Hs.444947 |
| TRIB2 | Hs.467751 |
| TRIB3 | Hs.516826 |
| TRIM24 | Hs.490287 |
| TRIM28 | Hs.467408 |
| TRIM33 | Hs.26837 |
| TRIO | Hs.130031 |
| TRPM6 | Hs.272225 |
| TRPM7 | Hs.512894 |
| TRRAP | Hs.203952 |
| TSSK1B | Hs.701555 |
| TSSK2 | Hs.694070 |
| TSSK3 | Hs.512763 |
| TSSK4 | Hs.314432 |
| TSSK6 | Hs.532711 |
| TTBK1 | Hs.485436 |
| TTBK2 | Hs.659846 |
| TTK | Hs.169840 |
| TTN | Hs.134602 |
| TXK | Hs.479669 |
| TYK2 | Hs.75516 |
| TYRO3 | Hs.381282 |
| UHMK1 | Hs.127310 |
| ULK1 | Hs.47061 |
| ULK2 | Hs.168762 |
| ULK3 | Hs.513034 |
| ULK4 | Hs.656192 |
| VRK1 | Hs.422662 |
| VRK2 | Hs.666703 |
| VRK3 | Hs.443330 |
| WEE1 | Hs.249441 |
| WEE2 | Hs.657927 |
| WNK1 | Hs.709894 |
| WNK2 | Hs.654856 |
| WNK3 | Hs.92423 |
| WNK4 | Hs.105448 |
| YES1 | Hs.194148 |
| YSK4 | Hs.659395 |
| ZAK | Hs.444451 |
| ZAP70 | Hs.234569 |
| PIK3C2A | Hs.175343 |
| PIK3C2B | Hs.497487 |
| PIK3C2G | Hs.22500 |
| PIK3C3 | Hs.464971 |
| PIK3CA | Hs.85701 |
| PIK3CB | Hs.239818 |
| PIK3CD | Hs.518451 |
| PIK3CG | Hs.32942 |
| PI4KA | Hs.529438 |
| PI4KB | Hs.632465 |
| PI4K2B | Hs.191701 |
| PI4K2A | Hs.25300 |
| AGK | Hs.699361 |
| CERK | Hs.200668 |
| DGKA | Hs.524488 |
| DGKB | Hs.567255 |
| DGKD | Hs.471675 |
| DGKE | Hs.239514 |
| DGKG | Hs.683449: |
| DGKH | Hs.659437 |
| DGKI | Hs.242947 |
| DGKQ | Hs.584858 |
| DGKZ | Hs.502461 |
| SPHK1 | Hs.68061 |
| SPHK2 | Hs.528006 |
| HRAS | Hs.37003 |
| KRAS | Hs.505033 |
| NRAS | Hs.486502 |
| PTEN | Hs.500466 |
| CDH1 | Hs.461086 |
| TP53 | Hs.654481 |
| CDKN2A | Hs.512599 |
| CDKN2B | Hs.72901 |
| APC | Hs.158932 |
| RB1 | Hs.408528 |
| CTNNB1 | Hs.476018 |
| BRCA1 | Hs.194143 |
| BRCA2 | Hs.34012 |
| NF1 | Hs.113577 |
| NF2 | Hs.187898 |
| GATA3 | Hs.524134 |
| MYC | Hs.202453 |
| INPP4A | Hs.469386 |
| PIK3R1 | Hs.132225 |
| PIK3R2 | Hs.371344 |
| PIK3R3 | Hs.655387 |
| PIK3R4 | Hs.149032 |
| PIK3R5 | Hs.278901 |
| PIK3R6 | Hs.255809 |
| CDC6 | Hs.405958 |
| CHD3 | Hs.25601 |
| COL1A1 | Hs.172928 |
| GAB1 | Hs.80720 |
| HAUS3 | Hs.665869 |
| IKBKB | Hs.597664 |
| IRS2 | Hs.442344 |
| IRS4 | Hs.460872 |
| KIAA1468 | Hs.465323 |
| KLHL4 | Hs.49075 |
| NFKB1 | Hs.654408 |
| NFKBIA | Hs.81328 |
| NFKBIE | Hs.458276 |
| PALB2 | Hs.444664 |
| RHEB | Hs.647068 |
| RNF220 | Hs.456557 |
| SNX4 | Hs.507243 |
| SP1 | Hs.649191 |
| USP28 | Hs.503891 |

# Supplementary Table 2: List of the 408 expressed kinase genes and their assignment into 10 kinase families.

| **Gene_Symbol** | **UniGene_ID** | **Kinase Group** |
| --- | --- | --- |
| AAK1 | Hs.468878 | Other |
| AATK | Hs.514575 | Other |
| ABL1 | Hs.431048 | TK |
| ABL2 | Hs.159472 | TK |
| ACTR2 | Hs.719274 | TKL |
| ADCK1 | Hs.413208 | Atypical |
| ADCK4 | Hs.130712 | Atypical |
| ADCK5 | Hs.283374 | Atypical |
| AKT1 | Hs.525622 | AGC |
| AKT2 | Hs.631535 | AGC |
| AKT3 | Hs.498292 | AGC |
| ALK | Hs.654469 | TK |
| ARAF | Hs.446641 | TKL |
| ATM | Hs.367437 | Atypical |
| ATR | Hs.271791 | Atypical |
| AURKA | Hs.250822 | Other |
| AURKB | Hs.442658 | Other |
| AURKC | Hs.98338 | Other |
| AXL | Hs.590970 | TK |
| BCKDK | Hs.513520 | Atypical |
| BLK | Hs.146591 | TK |
| BMP2K | Hs.146551 | TKL |
| BMPR1A | Hs.524477 | TKL |
| BMPR1B | Hs.598475 | TKL |
| BMPR2 | Hs.471119 | TKL |
| BMX | Hs.495731 | TK |
| BRAF | Hs.550061 | TKL |
| BRD2 | Hs.75243 | Atypical |
| BRD3 | Hs.522472 | Atypical |
| BRD4 | Hs.187763 | Atypical |
| BRDT | Hs.482520 | Atypical |
| BRSK1 | Hs.182081 | CAMK |
| BRSK2 | Hs.170819 | CAMK |
| BTK | Hs.159494 | TK |
| BUB1 | Hs.469649 | Other |
| BUB1B | Hs.631699 | Other |
| CAMK1 | Hs.434875 | CAMK |
| CAMK1D | Hs.659517 | CAMK |
| CAMK1G | Hs.199068 | CAMK |
| CAMK2A | Hs.716391 | CAMK |
| CAMK2B | Hs.351887 | CAMK |
| CAMK2D | Hs.144114 | CAMK |
| CAMK2G | Hs.523045 | CAMK |
| CAMK4 | Hs.591269 | CAMK |
| CAMKK1 | Hs.8417 | Other |
| CAMKK2 | Hs.297343 | Other |
| CASK | Hs.495984 | CAMK |
| CCRK | Hs.522274 | CMGC |
| CDC2 | Hs.334562 | CMGC |
| CDC2L2 | Hs.709182 | CMGC |
| CDC2L5 | Hs.233552 | CMGC |
| CDC2L6 | Hs.719138 | CMGC |
| CDC42BPA | Hs.35433 | CMGC |
| CDC42BPB | Hs.654634 | CMGC |
| CDC42BPG | Hs.293590 | CMGC |
| CDC7 | Hs.533573 | Other |
| CDK10 | Hs.699177 | CMGC |
| CDK2 | Hs.19192 | CMGC |
| CDK3 | Hs.706766 | CMGC |
| CDK4 | Hs.95577 | CMGC |
| CDK5 | Hs.647078 | CMGC |
| CDK6 | Hs.119882 | CMGC |
| CDK7 | Hs.184298 | CMGC |
| CDK8 | Hs.382306 | CMGC |
| CDK9 | Hs.150423 | CMGC |
| CDKL1 | Hs.679430 | CMGC |
| CDKL2 | Hs.591698 | CMGC |
| CDKL3 | Hs.105818 | CMGC |
| CDKL4 | Hs.403201 | CMGC |
| CDKL5 | Hs.659851 | CMGC |
| CLK1 | Hs.433732 | CMGC |
| CLK2 | Hs.73986 | CMGC |
| CLK3 | Hs.584748 | CMGC |
| CLK4 | Hs.406557 | CMGC |
| CSK | Hs.77793 | TK |
| DAPK1 | Hs.380277 | CAMK |
| DAPK2 | Hs.237886 | CAMK |
| DAPK3 | Hs.631844 | CAMK |
| DDR1 | Hs.631988 | TK |
| DDR2 | Hs.593833 | TK |
| DMPK | Hs.631596 | AGC |
| DYRK1A | Hs.719269 | CMGC |
| DYRK1B | Hs.130988 | CMGC |
| DYRK2 | Hs.173135 | CMGC |
| DYRK3 | Hs.164267 | CMGC |
| DYRK4 | Hs.439530 | CMGC |
| EEF2K | Hs.498892 | Atypical |
| EGFR | Hs.488293 | TK |
| EPHA1 | Hs.89839 | TK |
| EPHA10 | Hs.129435 | TK |
| EPHA2 | Hs.171596 | TK |
| EPHA3 | Hs.123642 | TK |
| EPHA4 | Hs.371218 | TK |
| EPHA5 | Hs.654492 | TK |
| EPHA6 | Hs.653244 | TK |
| EPHA7 | Hs.73962 | TK |
| EPHA8 | Hs.283613 | TK |
| EPHB1 | Hs.116092 | TK |
| EPHB2 | Hs.523329 | TK |
| EPHB3 | Hs.2913 | TK |
| EPHB4 | Hs.437008 | TK |
| EPHB6 | Hs.380089 | TK |
| ERBB2 | Hs.446352 | TK |
| ERBB3 | Hs.118681 | TK |
| ERBB4 | Hs.390729 | TK |
| FASTK | Hs.647094 | Atypical |
| FER | Hs.221472 | TK |
| FES | Hs.7636 | TK |
| FGFR1 | Hs.264887 | TK |
| FGFR2 | Hs.533683 | TK |
| FGFR3 | Hs.1420 | TK |
| FGFR4 | Hs.165950 | TK |
| FGR | Hs.1422 | TK |
| FLT1 | Hs.654360 | TK |
| FLT3 | Hs.507590 | TK |
| FLT4 | Hs.646917 | TK |
| FRK | Hs.89426 | TK |
| FYN | Hs.390567 | TK |
| GAK | Hs.369607 | Other |
| GCK | Hs.1270 | STE |
| GSK3A | Hs.466828 | CMGC |
| GSK3B | Hs.445733 | CMGC |
| HCK | Hs.655210 | TK |
| HIPK1 | Hs.532363 | CMGC |
| HIPK2 | Hs.397465 | CMGC |
| HIPK3 | Hs.201918 | CMGC |
| HIPK4 | Hs.79363 | CMGC |
| HUNK | Hs.109437 | CAMK |
| ICK | Hs.417022 | CMGC |
| IGF1R | Hs.643120 | TK |
| ILK | Hs.706355 | TKL |
| INSR | Hs.465744 | TK |
| INSRR | Hs.248138 | TK |
| IRAK1 | Hs.522819 | TKL |
| IRAK2 | Hs.449207 | TKL |
| IRAK3 | Hs.369265 | TKL |
| IRAK4 | Hs.138499 | TKL |
| ITK | Hs.558348 | TK |
| JAK1 | Hs.207538 | TK |
| JAK2 | Hs.656213 | TK |
| JAK3 | Hs.515247 | TK |
| KDR | Hs.479756 | TK |
| KIT | Hs.479754 | TK |
| KSR1 | Hs.133534 | TKL |
| KSR2 | Hs.375836 | TKL |
| LATS1 | Hs.716697 | AGC |
| LATS2 | Hs.78960 | AGC |
| LCK | Hs.470627 | TK |
| LIMK1 | Hs.647035 | TKL |
| LIMK2 | Hs.474596 | TKL |
| LMTK2 | Hs.444179 | TK |
| LMTK3 | Hs.207426 | TK |
| LRRK1 | Hs.407918 | TKL |
| LRRK2 | Hs.187636 | TKL |
| LTK | Hs.434481 | TK |
| LYN | Hs.699154 | TK |
| MAK | Hs.446125 | CMGC |
| MAP2K1 | Hs.145442 | STE |
| MAP2K2 | Hs.465627 | STE |
| MAP2K3 | Hs.514012 | STE |
| MAP2K4 | Hs.514681 | STE |
| MAP2K5 | Hs.114198 | STE |
| MAP2K6 | Hs.463978 | STE |
| MAP2K7 | Hs.531754 | STE |
| MAP3K1 | Hs.653654 | STE |
| MAP3K10 | Hs.466743 | STE |
| MAP3K11 | Hs.502872 | STE |
| MAP3K12 | Hs.713539 | STE |
| MAP3K13 | Hs.656069 | STE |
| MAP3K14 | Hs.404183 | STE |
| MAP3K15 | Hs.713701 | STE |
| MAP3K2 | Hs.145605 | STE |
| MAP3K3 | Hs.29282 | STE |
| MAP3K4 | Hs.390428 | STE |
| MAP3K5 | Hs.186486 | STE |
| MAP3K6 | Hs.194694 | STE |
| MAP3K7 | Hs.719192 | STE |
| MAP3K8 | Hs.432453 | STE |
| MAP3K9 | Hs.593542 | STE |
| MAP4K1 | Hs.95424 | STE |
| MAP4K2 | Hs.534341 | STE |
| MAP4K3 | Hs.655750 | STE |
| MAP4K4 | Hs.719073 | STE |
| MAP4K5 | Hs.130491 | STE |
| MAPK1 | Hs.431850 | CMGC |
| MAPK10 | Hs.125503 | CMGC |
| MAPK11 | Hs.57732 | CMGC |
| MAPK12 | Hs.432642 | CMGC |
| MAPK13 | Hs.178695 | CMGC |
| MAPK14 | Hs.485233 | CMGC |
| MAPK15 | Hs.493169 | CMGC |
| MAPK3 | Hs.861 | CMGC |
| MAPK4 | Hs.433728 | CMGC |
| MAPK6 | Hs.411847 | CMGC |
| MAPK7 | Hs.150136 | CMGC |
| MAPK8 | Hs.138211 | CMGC |
| MAPK9 | Hs.484371 | CMGC |
| MAPKAPK2 | Hs.643566 | CMGC |
| MAPKAPK3 | Hs.234521 | CMGC |
| MAPKAPK5 | Hs.413901 | CMGC |
| MARK1 | Hs.497806 | CAMK |
| MARK2 | Hs.567261 | CAMK |
| MARK3 | Hs.35828 | CAMK |
| MARK4 | Hs.34314 | CAMK |
| MAST1 | Hs.227489 | AGC |
| MAST2 | Hs.319481 | AGC |
| MAST3 | Hs.466184 | AGC |
| MAST4 | Hs.595458 | AGC |
| MASTL | Hs.276905 | AGC |
| MELK | Hs.184339 | CAMK |
| MET | Hs.132966 | TK |
| MLKL | Hs.119878 | TKL |
| MOS | Hs.533432 | Other |
| MST1R | Hs.517973 | STE |
| MUSK | Hs.521653 | TK |
| MYO3A | Hs.662630 | STE |
| MYO3B | Hs.671900 | STE |
| NEK1 | Hs.481181 | Other |
| NEK10 | Hs.506115 | Other |
| NEK11 | Hs.657336 | Other |
| NEK2 | Hs.153704 | Other |
| NEK3 | Hs.409989 | Other |
| NEK4 | Hs.631921 | Other |
| NEK5 | Hs.672144 | Other |
| NEK6 | Hs.197071 | Other |
| NEK7 | Hs.24119 | Other |
| NEK8 | Hs.448468 | Other |
| NEK9 | Hs.719118 | Other |
| NLK | Hs.208759 | CMGC |
| NRBP1 | Hs.515876 | Other |
| NRBP2 | Hs.521926 | Other |
| NRK | Hs.209527 | STE |
| NUAK1 | Hs.719171 | CAMK |
| NUAK2 | Hs.497512 | CAMK |
| OBSCN | Hs.656999 | CAMK |
| OXSR1 | Hs.475970 | STE |
| PAK1 | Hs.435714 | STE |
| PAK2 | Hs.518530 | STE |
| PAK3 | Hs.656789 | STE |
| PAK4 | Hs.20447 | STE |
| PAK6 | Hs.513645 | STE |
| PAK7 | Hs.32539 | STE |
| PASK | Hs.397891 | CAMK |
| PBK | Hs.104741 | Other |
| PDGFRA | Hs.74615 | TK |
| PDGFRB | Hs.509067 | TK |
| PDK1 | Hs.470633 | AGC |
| PDK2 | Hs.256667 | AGC |
| PDK3 | Hs.658190 | AGC |
| PDK4 | Hs.8364 | AGC |
| PHKG1 | Hs.715728 | CAMK |
| PHKG2 | Hs.196177 | CAMK |
| PIK3R4 | Hs.149032 | Other |
| PIM1 | Hs.81170 | CAMK |
| PIM2 | Hs.719294 | CAMK |
| PIM3 | Hs.530381 | CAMK |
| PINK1 | Hs.389171 | Other |
| PKN1 | Hs.466044 | AGC |
| PKN2 | Hs.440833 | AGC |
| PKN3 | Hs.300485 | AGC |
| PLK1 | Hs.592049 | Other |
| PLK2 | Hs.398157 | Other |
| PLK3 | Hs.632415 | Other |
| PLK4 | Hs.172052 | Other |
| PRKAA1 | Hs.43322 | AGC |
| PRKAA2 | Hs.437039 | AGC |
| PRKACA | Hs.631630 | AGC |
| PRKACB | Hs.487325 | AGC |
| PRKACG | Hs.158029 | AGC |
| PRKCA | Hs.531704 | AGC |
| PRKCB | Hs.460355 | AGC |
| PRKCD | Hs.155342 | AGC |
| PRKCE | Hs.580351 | AGC |
| PRKCG | Hs.631564 | AGC |
| PRKCH | Hs.333907 | AGC |
| PRKCI | Hs.478199 | AGC |
| PRKCQ | Hs.498570 | AGC |
| PRKCZ | Hs.496255 | AGC |
| PRKD1 | Hs.508999 | AGC |
| PRKD2 | Hs.466987 | AGC |
| PRKD3 | Hs.660757 | AGC |
| PRKDC | Hs.491682 | AGC |
| PRKG1 | Hs.654556 | AGC |
| PRKG2 | Hs.570833 | AGC |
| PRKX | Hs.390788 | AGC |
| PRKY | Hs.632287 | AGC |
| PSKH1 | Hs.513683 | CAMK |
| PSKH2 | Hs.680136 | CAMK |
| RAF1 | Hs.159130 | TKL |
| RET | Hs.350321 | TK |
| RIOK1 | Hs.437474 | Atypical |
| RIOK2 | Hs.27021 | Atypical |
| RIOK3 | Hs.719109 | Atypical |
| RIPK1 | Hs.519842 | TKL |
| RIPK2 | Hs.103755 | TKL |
| RIPK3 | Hs.268551 | TKL |
| RIPK4 | Hs.517310 | TKL |
| RNASEL | Hs.518545 | Other |
| ROCK1 | Hs.306307 | AGC |
| ROCK2 | Hs.591600 | AGC |
| ROR1 | Hs.654491 | TK |
| ROR2 | Hs.98255 | TK |
| ROS1 | Hs.1041 | TK |
| RYK | Hs.654562 | TK |
| SBK1 | Hs.97837 | Other |
| SBK2 | Hs.532676 | Other |
| SCYL1 | Hs.238839 | Other |
| SCYL2 | Hs.506481 | Other |
| SCYL3 | Hs.435560 | Other |
| SGK1 | Hs.510078 | AGC |
| SGK196 | Hs.491646 | AGC |
| SGK2 | Hs.300863 | AGC |
| SGK269 | Hs.9587 | AGC |
| SGK3 | Hs.613417 | AGC |
| SGK493 | Hs.408542 | AGC |
| SIK1 | Hs.282113 | CAMK |
| SIK2 | Hs.269128 | CAMK |
| SIK3 | Hs.167451 | CAMK |
| SLK | Hs.591922 | STE |
| SMG1 | Hs.460179 | Atypical |
| SNRK | Hs.476052 | CAMK |
| SPEG | Hs.21639 | CAMK |
| SRC | Hs.195659 | TK |
| SRM | Hs.76244 | TK |
| SRMS | Hs.411061 | TK |
| SRPK1 | Hs.443861 | CMGC |
| SRPK2 | Hs.285197 | CMGC |
| SRPK3 | Hs.104865 | CMGC |
| STK10 | Hs.719134 | CAMK |
| STK11 | Hs.515005 | CAMK |
| STK16 | Hs.153003 | CAMK |
| STK17A | Hs.709489 | CAMK |
| STK17B | Hs.88297 | CAMK |
| STK19 | Hs.654371 | CAMK |
| STK24 | Hs.508514 | CAMK |
| STK25 | Hs.516807 | CAMK |
| STK3 | Hs.492333 | CAMK |
| STK31 | Hs.309767 | CAMK |
| STK32A | Hs.585069 | CAMK |
| STK32B | Hs.133062 | CAMK |
| STK32C | Hs.469002 | CAMK |
| STK33 | Hs.501833 | CAMK |
| STK35 | Hs.100057 | CAMK |
| STK36 | Hs.471404 | CAMK |
| STK38 | Hs.409578 | CAMK |
| STK38L | Hs.184523 | CAMK |
| STK39 | Hs.276271 | CAMK |
| STK4 | Hs.472838 | CAMK |
| STK40 | Hs.471768 | CAMK |
| SYK | Hs.371720 | TK |
| TAF1 | Hs.158560 | Atypical |
| TAF1L | Hs.591086 | Atypical |
| TBCK | Hs.292986 | Other |
| TBK1 | Hs.505874 | Other |
| TEC | Hs.479670 | TK |
| TESK1 | Hs.708096 | TKL |
| TESK2 | Hs.591499 | TKL |
| TGFBR1 | Hs.494622 | TKL |
| TGFBR2 | Hs.82028 | TKL |
| TIE1 | Hs.78824 | TK |
| TLK1 | Hs.719163 | Other |
| TLK2 | Hs.445078 | Other |
| TNIK | Hs.34024 | STE |
| TNK1 | Hs.203420 | TK |
| TNK2 | Hs.518513 | TK |
| TRIM24 | Hs.490287 | Atypical |
| TRIM28 | Hs.467408 | Atypical |
| TRIM33 | Hs.26837 | Atypical |
| TRIO | Hs.130031 | CAMK |
| TRRAP | Hs.203952 | Atypical |
| TSSK1B | Hs.701555 | CAMK |
| TSSK2 | Hs.694070 | CAMK |
| TSSK3 | Hs.512763 | CAMK |
| TSSK4 | Hs.314432 | CAMK |
| TSSK6 | Hs.532711 | CAMK |
| TTBK1 | Hs.485436 | CK1 |
| TTBK2 | Hs.659846 | CK1 |
| TTK | Hs.169840 | Other |
| TTN | Hs.134602 | CAMK |
| TXK | Hs.479669 | TK |
| TYK2 | Hs.75516 | TK |
| TYRO3 | Hs.381282 | TK |
| UHMK1 | Hs.127310 |  |
| ULK1 | Hs.47061 | Other |
| ULK2 | Hs.168762 | Other |
| ULK3 | Hs.513034 | Other |
| ULK4 | Hs.656192 | Other |
| VRK1 | Hs.422662 | CK1 |
| VRK2 | Hs.666703 | CK1 |
| VRK3 | Hs.443330 | CK1 |
| WEE1 | Hs.249441 | Other |
| WEE2 | Hs.657927 | Other |
| WNK1 | Hs.709894 | Other |
| WNK2 | Hs.654856 | Other |
| WNK3 | Hs.92423 | Other |
| WNK4 | Hs.105448 | Other |
| YES1 | Hs.194148 | TK |
| YSK4 | Hs.659395 | STE |
| ZAK | Hs.444451 | TKL |
| ZAP70 | Hs.234569 | TK |
| CDKN2A | Hs.512599 | CMGC |
| CDKN2B | Hs.72901 | CMGC |
| PIK3R1 | Hs.132225 | Other |
| PIK3R2 | Hs.371344 | Other |
| PIK3R3 | Hs.655387 | Other |
| PIK3R4 | Hs.149032 | Other |
| PIK3R5 | Hs.278901 | Other |
| PIK3R6 | Hs.255809 | Other |

# Supplementary Table 5: Results for comparison of the mean mutational load per patient per gene between 10 kinase groups. All p-values were computed using an unpaired Wilcoxon test, and were adjusted for multiple testing using the Bonferroni correction.

| **Kinase Group[[1]](#footnote-2)** | **P-value _(All variants) Primary vs Metastatic_** | **P-value _(HFI variants) Primary vs Metastatic_** | **P-value (HFI variants + Expressed Kinases) Primary vs Metastatic** | **P-value _(ER-positive vs HER2-positive)_** | **P-value _(ER-positive vs TNBC)_** | **P-value _(TNBC vs HER2-positive)_** |
| --- | --- | --- | --- | --- | --- | --- |
| AGC | 0.71 | 0.42 | 0.56 | 0.37 | 0.72 | 0.41 |
| Atypical | 0.76 | 0.48 | 0.65 | 0.64 | 0.4 | 0.33 |
| CAMK | 0.73 | 0.09 | 0.34 | 0.83 | 0.6 | 0.68 |
| CK1 | 0.25 | 0.63 | 0.84 | 0.39 | 0.48 | 0.54 |
| CMGC | 0.25 | 0.06 | 0.25 | 0.52 | 0.72 | 0.39 |
| Other | 0.81 | 0.89 | 0.68 | 0.30 | 0.82 | 0.37 |
| RGC | 0.96 | 0.24 | 0.34 | 0.60 | 0.80 | 0.51 |
| STE | 0.19 | 0.49 | 0.24 | 0.937 | 0.20 | 0.39 |
| TK | 0.23 | 0.06 | 0.54 | **0.04** | 0.40 | **0.01** |
| TKL | **0.02** | 0.16 | **0.04** | 0.26 | 0.68 | 0.13 |

1. Patients were categorized into different subtypes, and the mutation data was filtered for expressed kinases and predicted HFI>2.5. [↑](#footnote-ref-2)
